# Gene expression pattern of adenosine receptors in lung tumors

**DOI:** 10.1002/cnr2.1747

**Published:** 2022-10-25

**Authors:** Elnaz Asgharkhah, Marie Saghaeian Jazi, Jahanbakhsh Asadi, Seyyed Mehdi Jafari

**Affiliations:** ^1^ Metabolic Disorders Research Center Golestan University of Medical Sciences Gorgan Iran; ^2^ Department of Biochemistry and Biophysics, Faculty of Medicine Golestan University of Medical Sciences Gorgan Iran

**Keywords:** adenosine receptors, lung cancer, real time PCR, receptor expression

## Abstract

**Background:**

Adenosine, a purine nucleoside, plays an important function in the pathogenesis of cancer through interaction with the cell surface G protein‐coupled adenosine receptors. It is important to determine the expression pattern of these receptors in different cancers. Previously in our lab, we found up‐regulation of A1 adenosine receptor (AR) in lung tumors playing as a putative target for cancer cell inhibition, and here we aimed to investigate the significance of other adenosine receptor isoforms (A2aAR, A2bAR, and A3AR).

**Methods:**

In this study, first of all, we evaluated the adenosine receptors gene expression in the bioinformatics database (GENT2). Then the genes expression was measured experimentally in the 20 lung cancer tumor tissues in comparison to the matched tumor‐adjacent normal tissue (as control). The mRNA expression of receptors was evaluated by real‐time PCR. The tumors were categorized by the tumor size and the gene expression change was evaluated.

**Results:**

The experimental results indicated a significant increase in A2aAR (*p* value = .021) and A3AR (*p* value = .01) expression in lung tumor tissues compared to the adjacent tumor margins which were in accordant to bioinformatics analysis. We found a non‐significant increase in A2bAR expression; however, when comparing the patients according to the tumor size, our data showed that the expression of A2bAR adenosine receptor in patients with smaller lung tumor sizes was higher than the other group (*p* = .011).

**Conclusion:**

The results of this study showed that adenosine receptors A3AR, and A2aAR are highly expressed in lung tumors relative to tumor‐adjacent normal tissue. We suggest that overexpression of adenosine receptors in lung cancer is due to their regulatory role in various aspects of lung cancer.

## 1 INTRODUCTION

Lung cancer remains the leading cause of cancer death in the world.^1^, ^2^ Around 95% of lung cancers are classified into two main types of non‐small cell lung cancer (NSCLC) and small cell lung cancer (SCLC).^3^ The SCLC is a high‐grade neuroendocrine cancer that causes approximately 15% of all lung cancers and over 200 000 deaths worldwide each year.^4^ More than 85% of all cases of lung cancer are NSCLC.^5^ The most frequent subtypes of NSCLC are adenocarcinoma, squamous cell carcinoma, and large cell lung carcinoma.^6^ Despite using advanced anti‐cancer therapeutic strategies, death from lung cancer has increased recently.^7^

Lung cancer is a multifactorial complex disease and has a genetic alteration background. Studying the gene aberrations and mutations associated with lung cancer can impact the molecular therapy selection and it also can help to increase our current knowledge of drug resistance.^8^ Personalized medicine can help some patients suffering from lung cancer with specific gene mutations like epithermal growth factor receptor (EGFR). Studying the genes involved in lung cancer progression may help to find potential molecular targets to develop novel therapeutics strategies for lung cancer in future.^9^

Recently, the adenosine receptors signaling pathway have been introduced as emerging cancer associated metabolic pathways with a dys‐regulated expression of adenosine receptors in different cancers.^10^ Adenosine is a nucleoside produced by de‐phosphorylation of adenine nucleotides in the body and modulate various physiologic function.^11^ It plays an important role in the pathogenesis of cancer initiation and promotion through interaction with cell surface G protein‐coupled adenosine receptors (AR), namely, P1 receptors (P1Rs).^12^, ^13^ These receptors consist of four different subtypes of A1AR, A2aAR, A2bAR, and A3AR.^14^ Activation or inhibition of the adenosine receptors using agonists or antagonist consequently will result various cellular functions through cyclic adenosine monophosphate (cAMP) regulation by different signaling pathways.^15^ The A1 and A3 are coupled to G_i/o_ protein, which can inhibit adenylate cyclase (AC) thereby decreasing cAMP.^16^, ^17^ In contrast to A2aAR, the A2bAR couples to G_s/olf_ protein, which stimulates the production of cAMP and increases PKA signaling.^18^

Adenosine receptors are involved in many crucial processes in cancer including regulation of apoptosis, proliferation, metastasis and angiogenesis.^19^ Since the targeting of adenosine receptors and adenosine signaling is currently being tested in clinical trials,^20^, ^21^, ^22^ studying adenosine receptors expression in various cancers is of a certain importance to help identifying cancer subtypes more likely to respond adenosine‐targeting agents. Numerous studies in recent years have revealed overexpression of adenosine receptors in various cancers. However, the role of adenosine receptors in cell growth and apoptosis is controversial, as these receptors have different effect depending on the type of tissue, which they are expressed.^23^

We previously reported a significant up‐regulation of the A1 adenosine receptor in lung cancer tumors. In addition, we found in‐vitro inhibition of the A1AR can induce lung cancer cell death^24^; indicating its importance in lung cancer progression. Then here we aimed to investigate the expression of the other adenosine receptor isoforms in lung cancer.

## 2 MATERIAL

### 2.1 Bioinformatics

For bioinformatics analysis, the microarray data available from the GENT2 database (http://gent2.appex.kr/gent2/) was downloaded for each gene.^25^ The Log2 fold changes were visualized using the graph pad prism software.

### 2.2 Tumor tissues

In this study, a collection of 20 lung cancer tumor tissues including 14 of adenocarcinoma and 6 of squamous cell carcinoma were used. Patients were 5 females and 15 males with a mean age of 57.16 years old. For control, the matched tumor adjacent normal tissue from each patient was used. The TNM (T: tumor size, N: spread to the nearby lymph nodes; and M: metastasis) stage of tumors were collected according to the pathology results and were used for data analysis. Briefly, half of the samples were TNM II (50%) and the other half were TNM I (20%) or TNM > II (30%). This study was approved by the Ethical committee of the Golestan University of Medical Sciences (Approval code: IR. GOUMS.REC.1398.344) and written consent was taken from all the patients.

### 2.3 RNA extraction

Tumor or matched control tissues were powdered using liquid nitrogen. Then the RNA was extracted using Trizol reagent (Thermo Fisher, Catalog number: 15596026) following the manufacturer's recommended protocol. To avoid possible DNA contamination, all RNA samples were treated with DNaseI (Thermo Fisher, Catalog Number: EN0525). Then the equal amount of total RNA (1000 ng) of each sample was used for first strand cDNA synthesis using reverse transcriptase (Yekta Tajhiz, Iran, Catalog Number: YT4500).

### 2.4 Real time PCR

The genes expression level was measured using the SYBR green real time‐PCR method (Yekta Tajhiz, Iran, Catalog Number: YT2551) in ABI system 7300. The thermal cycling condition was as follows: initial denaturation at 94°C for 2 min, 40 cycles of denaturation at 94°C for 30 s, annealing at 60°C for 45 s and polymerization at 72°C for 75 s. The specificity of product amplification was checked by final melt curve analysis. As the housekeeping gene for normalization, the gene expression of GAPDH was measured. The primers of the A2aAR, A2bAR, A3AR receptor and GAPDH genes are listed as following: A2a adenosine receptor F: CGCTCCGGTACAATGGCTT; A2a adenosine receptor R: TTGTTCCAACCTAGCATGGGA, A2b adenosine receptor F: GGGGTGGAACAGTAAAGACAG; A2b adenosine receptor R: CAGCAGCTTTCATTCGTGGTT and A3 adenosine receptor F: GTGCTGGTCATGCCTTTGG; A3 adenosine receptor R: CGTGGGTAAAGATAAGCAGTAGG. The 2^−ddct^ formula was used for gene expression semi‐quantitative comparison.

### 2.5 Statistical analysis

In the current study, the *p* value of less than .05 was considered statistically significant. The studied variables were analyzed using SPSS 20 software. The normality of data was checked and then the Mann–Whitney *U* non‐parametric test was used to analyze the results of gene expression in tumor samples compared to normal adjacent tissue. To analyze the correlation between genes expression we used Spearman correlation test.

## 3 RESULT

**FIGURE 1:**
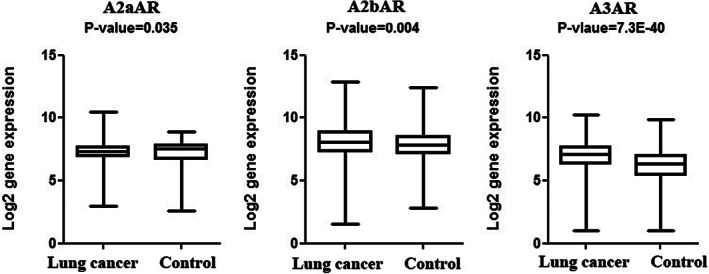
Evaluation of log2 gene expression of adenosine receptors downloaded from GENT2 database. A box plot (whiskers: min to max) representation of log 2 gene expression of microarray data (GPL570 platform HG‐U133‐plus‐2)

The genes expression of lung tumor tissues in comparison to lung normal tissues was analyzed from the microarray data available from the GNET2 database. As shown in Figure 1, there was a significant up‐regulation for all adenosine receptor genes in tumor tissues (Lung tumor *N* = 3262, Lung normal *N* = 508).

**FIGURE 2:**
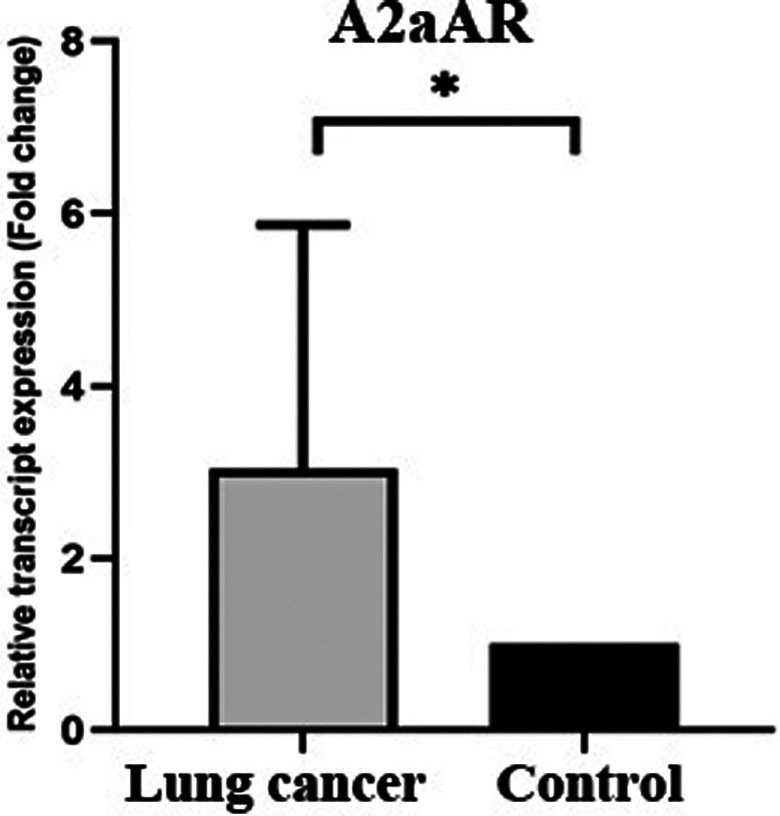
Evaluation of relative expression of adenosine A2aAR gene in lung tumor tissue and tumor margin. Adenosine A2aAR expression was assessed using real‐time PCR in tumor samples and tumor margins and a significant level of less than .05 was assumed. Adenosine A2AAR expression increased in tumor tissue compared to control and this increase was statistically significant (*p‐value <.05)

The gene expression level of adenosine receptors (A2aAR, A2bAR, and A3AR) in lung cancer tissues (20 cases) and tumor margin tissues (20 cases) was examined by real‐time PCR experiments. Our finding showed that the mean relative expression of the A2aAR (Figure 2) was significantly increased around 2.5 times in lung tumor tissues compared to the normal adjacent tissue as control (Mann–Whitney *U* test, *p* value = .021).

**FIGURE 3:**
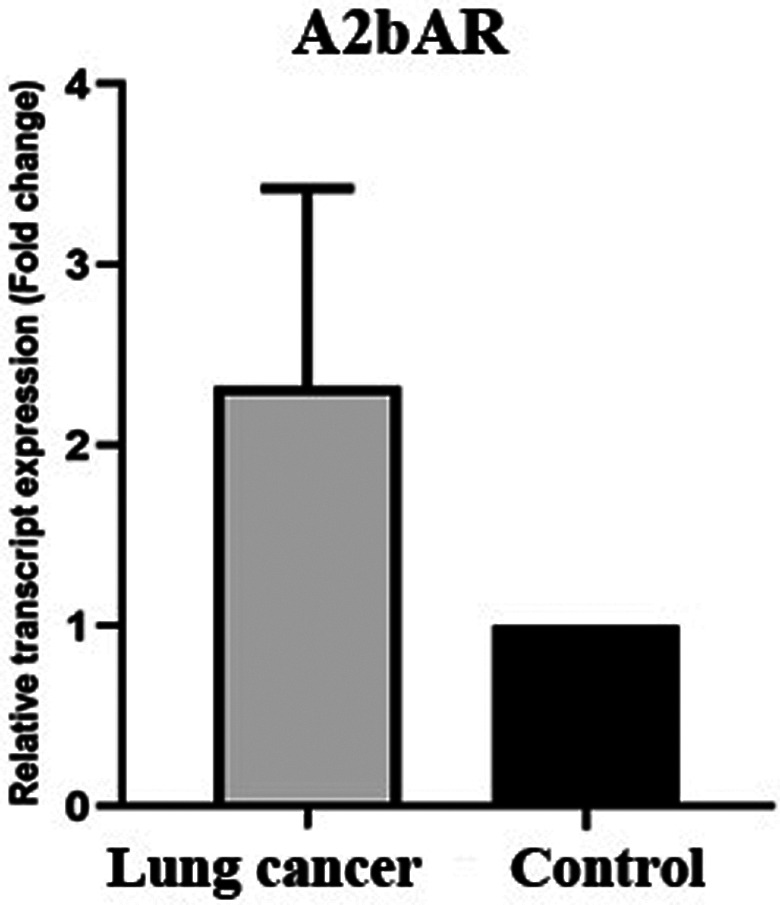
Evaluation of relative expression of A2bAR gene in lung tumor tissue and tumor margin. Adenosine receptor expression was assessed using real‐time PCR in tumor samples and tumor margins and a significance level of less than .05 was considered. Adenosine A2BAR expression was increased in tumor tissue compared to control, but this increase was not statistically significant (*p*‐value >.05)

Our data also showed the expression of adenosine A2bAR in tumor tissues was around 2 times higher than the normal adjacent tissue (Figure 3). But this increase was not significant (Mann–Whitney *U* test, *p* value = .056).

**FIGURE 4:**
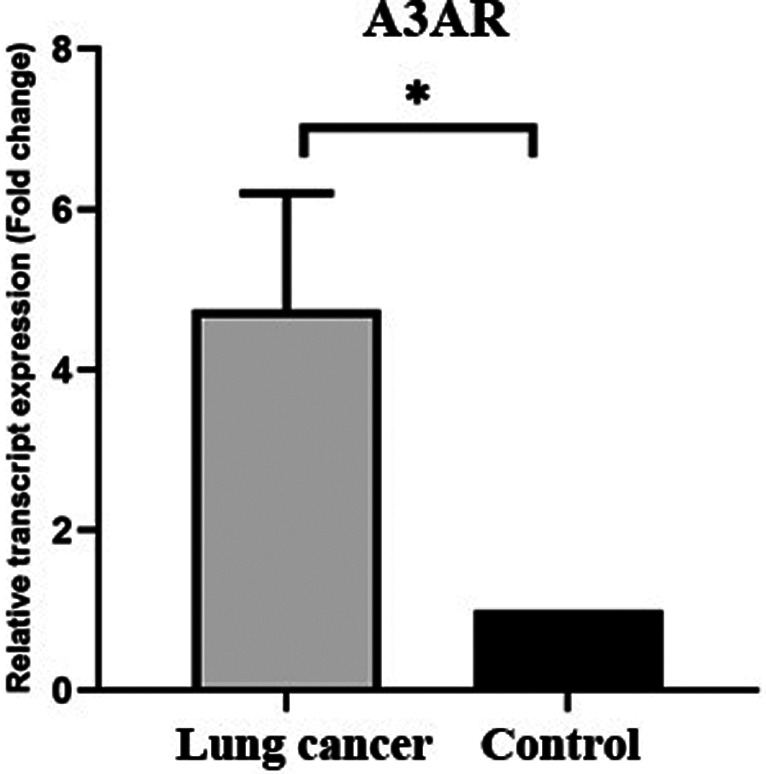
Evaluation of relative expression of adenosine A3AR gene in lung tumor tissue and tumor margin. Adenosine A3AR expression was assessed using real‐time PCR in tumor samples and tumor margins and a significant level of less than .05 was assumed. Adenosine A3AR expression increased in tumor tissue compared to control and this increase was statistically significant (*p*‐value <.05)

Furthermore, the results showed (Figure 4) that lung tumor tissues over‐ express mRNA of the A3AR significantly over 4 times in comparison to the normal adjacent tissue (Mann–Whitney *U* test, *p* value = .01). The correlation analysis showed (Table 1) that there is a significant direct correlation between the expression level of A2bAR and A3AR (Spearman correlation *R* = .818, *p* = .000).

**TABLE 1:** Correlation between expression of adenosine receptor in lung tumor

Correlation	*r*	*p* value
A2aAR and A2bAR	−.118	.653
A2aAR and A3AR	−.156	.536
A2bAR and A3AR	.818	.000

**FIGURE 5:**
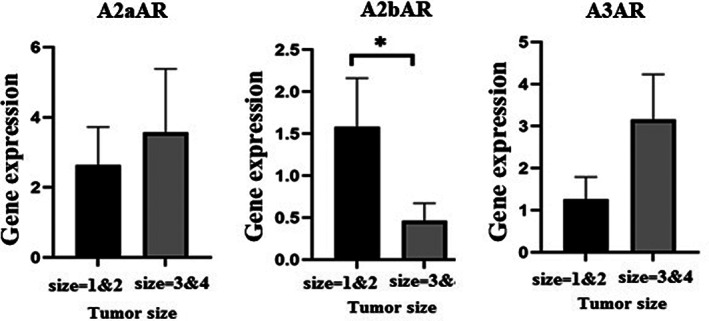
Evaluation of the adenosine receptor expression base on tumor size. The size of the tumor was categorized into two groups: one (between 1 and 2 cm) and two (between 3 and 4 cm). Our data showed that the expression of A2bAR in patients with smaller lung tumor sizes was higher than the other group (*p* = .011)

For more investigation, the size of the tumor was categorized into two groups: one (between 1 and 2 cm) and two (between 3 and 4 cm). Our data showed (Figure 5) that the expression of A2bAR in patients with smaller lung tumor sizes was higher than the other group (*p* = .011). For the other pathological features of the tumor including involved lymph nodes and distant metastasis, no significant correlation was found.

## 4 DISCUSSION

Adenosine plays important functions in many cellular pathways including cell growth and apoptosis of different cell types of epithelial, neuronal, endothelial and immune response cells.^2^ Rather than its normal physiological role, there is emerging evidence supporting the role of adenosine or adenosine receptor in the proliferation, viability, and migration of the tumor cells in different cancers.^16^, ^26^, ^27^ In this study, the expression of the adenosine receptors (A2a, A2b, and A3) at RNA level was measured in the lung cancer tissues in comparison with normal adjacent tissue by real‐time PCR. Our data indicated a significant increase in A2aAR expression in lung tumor tissues. Similar findings were reported in the literature in lung,^28^ prostate,^12^ and colorectal^29^ tumors. We also found increased expression of the A2bAR in the lung tumor tissues compared to adjacent normal tissue, consistent with study of Sui et al.^30^ in lung adenocarcinoma, Ma et al.^31^ in colon cancer and Mousavi et al.^12^ in prostate cancer. Finally, this study showed a significant elevation in A3AR in lung tumor tissues. Similar results were previously reported in other tumor types including prostate cancer^12^ and colon cancer^32^ for A3AR gene expression.

Over‐expression of adenosine receptor in tumor tissues may suggests a potential oncogenic function; however when considering the role of adenosine, contradictory effects in different cancers have been reported. Several in vitro experiments indicated adenosine modulate adenosine receptors, with either stimulatory or inhibitory effects on cell growth.^33^ In some cancers like ovarian,^34^ and prostate^35^ cancers; studies illustrated cancer inhibitory effect for adenosine. In contrast, another study reported that adenosine promotes cellular proliferation and migration in breast cancer through activation of the A2bAR.^16^

The overexpression of adenosine receptors reported in various cancers provides novel therapeutic possibilities in cancer treatment. Currently, different clinical trials are investigating the anti‐cancer potential of adenosine receptors modulation in cancer treatment (NCT05024097 and NCT04381832). Also in our previous study, we found the anti‐cancer effect of adenosine receptor antagonist in lung cancer cell line in vitro^24^; however, more studies are necessary to investigate the potential of adenosine receptor targeting for lung cancer treatment.

## 5 CONCLUSION

The results of this study show that the A3AR and A2aAR adenosine receptors are highly expressed in lung tumor tissues relative to tumor adjacent normal tissue. Considering the expression of these receptors in lung cancer cells, it can be concluded that adenosine receptors have an important role in lung cancer and could be considered as a potential therapeutic target in lung cancer.

## AUTHOR CONTRIBUTIONS

**Elnaz Asgharkhah:** Investigation (equal); methodology (equal); writing – original draft (equal). **Marie Saghaeian Jazi:** Formal analysis (equal); methodology (equal); writing – review and editing (equal). **Jahanbakhsh Asadi:** Conceptualization (equal); writing – review and editing (equal). **Seyyed Mehdi Jafari:** Conceptualization (equal); supervision (equal); writing – review and editing (equal).

## CONFLICT OF INTEREST

The authors have stated explicitly that there are no conflicts of interest in connection with this article.

## ETHICS STATEMENT

This study was approved by the Clinical Research Ethics Committee of Golestan University of Medical Sciences (ethical code: IR. GOUMS.REC.1398.344).

## Data Availability

The data that support the findings of this study are available from the corresponding author upon reasonable request.
